# UPLC-ESI-MS/MS Profiling and Cytotoxic, Antioxidant, Anti-Inflammatory, Antidiabetic, and Antiobesity Activities of the Non-Polar Fractions of *Salvia hispanica* L. Aerial Parts

**DOI:** 10.3390/plants12051062

**Published:** 2023-02-27

**Authors:** Afaf E. Abdel Ghani, Muneera S. M. Al-Saleem, Wael M. Abdel-Mageed, Ehsan M. AbouZeid, Marwa Y. Mahmoud, Rehab H. Abdallah

**Affiliations:** 1 Pharmacognosy Department, Faculty of Pharmacy, Zagazig University, Zagazig 44519, Egypt; 2 Department of Chemistry, Science College, Princess Nourah Bint Abdulrahman University, P.O. Box 84428, Riyadh 11671, Saudi Arabia; 3 Department of Pharmacognosy, College of Pharmacy, King Saud University, P.O. Box 2457, Riyadh 11451, Saudi Arabia; 4 Department of Pharmacognosy, Faculty of Pharmacy, Assiut University, Assiut 71526, Egypt

**Keywords:** *Salvia hispanica*, chia, Lamiaceae, UPLC-ESI-MS/MS, omega-3 fatty acid, cytotoxic, antioxidant, antiobesity

## Abstract

*Salvia hispanica* L. is an annual herbaceous plant commonly known as “Chia”. It has been recommended for therapeutic use because of its use as an excellent source of fatty acids, protein, dietary fibers, antioxidants, and omega-3 fatty acids. A literature survey concerning phytochemical and biological investigations of chia extracts revealed less attention towards the non-polar extracts of *S. hispanica* L. aerial parts, which motivates us to investigate their phytochemical constituents and biological potentials. The phytochemical investigation of the non-polar fractions of *S. hispanica* L. aerial parts resulted in the tentative identification of 42 compounds using UPLC-ESI-MS/MS analysis with the isolation of β-sitosterol (**1**), betulinic acid (**2**), oleanolic acid (**3**), and β-sitosterol-3-*O*-β-D-glucoside (**4**). GLC-MS analysis of the seeds’ oil showed a high concentration of omega-3 fatty acid, with a percentage of 35.64% of the total fatty acid content in the seed oil. The biological results revealed that the dichloromethane fraction showed promising DPPH radical-scavenging activity (IC_50_ = 14.73 µg/mL), antidiabetic activity with significant inhibition of the α-amylase enzyme (IC_50_ 673.25 μg/mL), and anti-inflammatory activity using in vitro histamine release assay (IC_50_ 61.8 μg/mL). Furthermore, the dichloromethane fraction revealed moderate cytotoxic activity against human lung cancer cell line (A-549), human prostate carcinoma (PC-3), and colon carcinoma (HCT-116) with IC_50s_ 35.9 ± 2.1 μg/mL, 42.4 ± 2.3 μg/mL, and 47.5 ± 1.3 μg/mL, respectively, and antiobesity activity with IC_50_ 59.3 μg/mL, using pancreatic lipase inhibitory assay. In conclusion, this study’s findings not only shed light on the phytochemical constituents and biological activities of the non-polar fractions of chia but also should be taken as a basis for the future in vivo and clinical studies on the safety and efficacy of chia and its extracts. Further study should be focused towards the isolation of the active principles of the dichloromethane fraction and studying their efficacy, exact mechanism(s), and safety, which could benefit the pharmaceutical industry and folk medicine practitioners who use this plant to cure diseases.

## 1. Introduction

The Lamiaceae (Labiatae, Mint) family comprises 245 genera and about 7886 species worldwide. Many genera belonging to this family have important uses in medicine, the culinary arts, and cosmetics [1]. The chemical components of the family members have biological roles with therapeutic value; these chemicals include essential oils, alkaloids, flavonoids, glycosides, steroids, coumarins, tannins, and terpenoids [2].

*Salvia hispanica* L. is an annual herb that is commonly known as “Chia”, native to southern Mexico and northern Guatemala [3]. *Salvia hispanica* L. is mainly grown for its seeds, which are widely consumed because of their high nutritional and medicinal value [4,5,6,7,8,9]. Globally, research has been conducted investigating the benefits of chia seeds and oil and their applications in the food, cosmetic, medical, and pharmaceutical industries. A literature survey revealed more concern towards chia seeds’ constituents and biological activities, with less attention to other parts of the plant. Previous phytochemical analyses of *S. hispanica* seeds’ constituents indicated the presence of flavonoids and phenolic acids that are linked to their antioxidant, antiobesity, antidiabetic, and antimicrobial activities [4,5,6,7,8,9,10,11,12,13]. In contrast, only a few studies have reported on the phytochemical and biological activities of *S. hispanica* L. aerial parts, which exhibit the presence of neoclerodane-type diterpenoids with the tentative identification of different phenolic compounds [14,15,16].

To the best of our knowledge, there are no bibliographic data in the literature about the phytochemical composition and biological activities of the aerial parts of *S. hispanica* cultivated in Egypt except our previous work that focused on the investigation of the main bioactive constituents of the polar fraction of the aerial parts, which resulted in the tentative detection of 37 compounds, using UPLC-ESI-MS/MS analysis with the isolation of 1,2,4,5 tetrahydroxy benzene, leucantho flavone, and rhamnetin [17]. The current study focused on the identification of the active constituents of the non-polar fractions of the aerial parts of *S. hispanica* cultivated in Egypt with the investigation of their potential biological activities, including cytotoxic, antioxidant, anti-inflammatory, antidiabetic, and antiobesity activities, to attract attention and provide evidence for their therapeutic value.

## 2. Results and Discussion

### 2.1. Structural Identification of Constituents by UPLC-ESI-MS/MS

**Figure 1:**
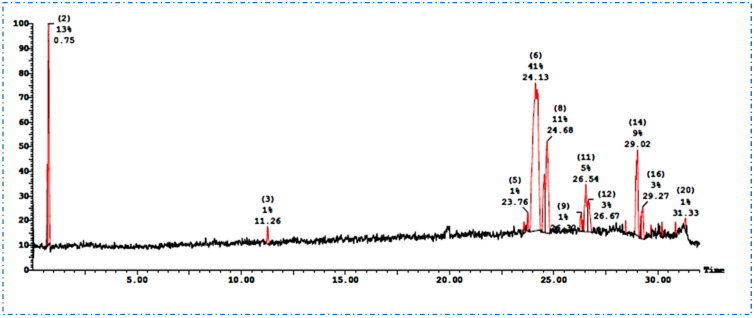
UPLC-ESI-MS/MS chromatogram of the light petroleum fraction of *S. hispanica* L. aerial parts.

UPLC-ESI-MS/MS in positive ionization mode was used to analyze the light petroleum fraction of *S. hispanica* L. aerial parts (Figure 1). The tentative detection of nine compounds was based on the fragmentation patterns that were compared with the available literature data, as shown in Table 1.

Compound **1** (Rt, 23.57) showed a molecular ion peak [M+H]^+^ at *m*/*z* 577, a base peak [M]^+^ at *m*/*z* 576, as well as a fragment ion at *m*/*z* 415 [M+H-Glu]^+^. In accordance with this fragmentation pattern, the compound was classified as β-sitosterol-3-*O*-β-D-glucoside [18]. 

Compound **2** (Rt, 23.76) showed a precursor ion [M+H]^+^ at *m*/*z* 301 as well as a fragment ion at *m*/*z* 227 [M+H-propene unit-H_2_O-CH_2_]^+^. By this fragmentation pattern, the compound was classified as sugiol [19]. Compound **3** (Rt, 23.77) showed a precursor ion [M+H]^+^ at *m*/*z* 279 as well as fragment ions at *m*/*z* 301 [M+Na]^+^, 279 [M+H]^+^,and 261 [M+H-H_2_O]^+^. The compound (**3**) was identified as 7α-hydroxy-14,15-dinorlabd-8(17)-en-13-one based on this fragmentation [20]. 

Compound **4** (Rt, 24.13) showed a molecular ion peak [M]^+^ at *m*/*z* 414 and a fragment ion at *m*/*z* 396 [M-H_2_O]^+^. In accordance with this fragmentation pattern, the compound was classified as β-sitosterol [21].

Compounds **5**, **6,** and **9** (Rt, 24.68, 26.54 & 29.68 min) revealed protonated molecular ions at *m*/*z* 281, 279, and 257, respectively. These fragments were in good agreement with the characteristics of linoleic acid, linolenic acid, and palmitic acid, respectively. These fatty acids were previously detected in other *salvia* species [22].

Compound **7** (Rt 29.68 min) showed a molecular ion fragment at *m*/*z* 457 [M+H]^+^ and was tentatively identified as betulinic acid. The HPLC-ESI-MS spectra of this compound showed MS^2^ fragment ions at *m*/*z* 248 [C_16_H_24_O_2_]^+^, 203 [248-COOH]^+^, 207 [M-C_16_H_27_]^+^, 189 [207-H_2_O]^+^,and 175,which comprise the characteristic fragments for betulinic acid [23].

In the same manner, compound **8** (Rt, 29.68 min) showed a molecular ion fragment at *m*/*z* 457 [M+H]^+^ and prominent ion fragments at *m*/*z* 248 and 207 [C_14_H_23_O]^+^; it also showed a fragment ion at 203 [C_15_H_23_]^+^,because of loss of COOH from 248,and another fragment ion at *m*/*z* 189 [207-H_2_O]^+^.This fragmentation pattern was in good agreement with the previous report of oleanolic acid [24].

**Figure 2:**
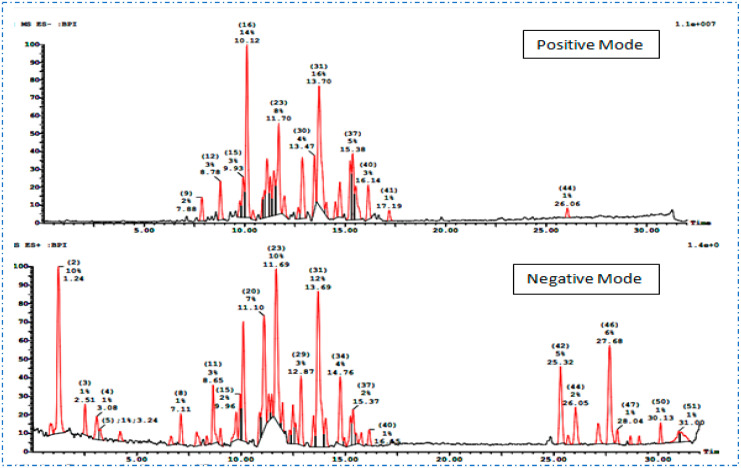
Chromatograms of UPLC-ESI-MS/MS in positive and negative modes of the dichloromethane fraction of *S. hispanica* L. aerial parts.

For the dichloromethane fraction, the UPLC-ESI-MS/MS in negative and positive ion modes led to the identification of 33 compounds (Figure 2). The compounds were arranged according to retention time (R_t_) and classified accordingly into different classes including phenolic acids, flavonoids, diterpenoids, alkaloids, tannins, steroids, triterpenoids, fatty acids, and miscellaneous compounds (Table 2).

**Figure 3:**
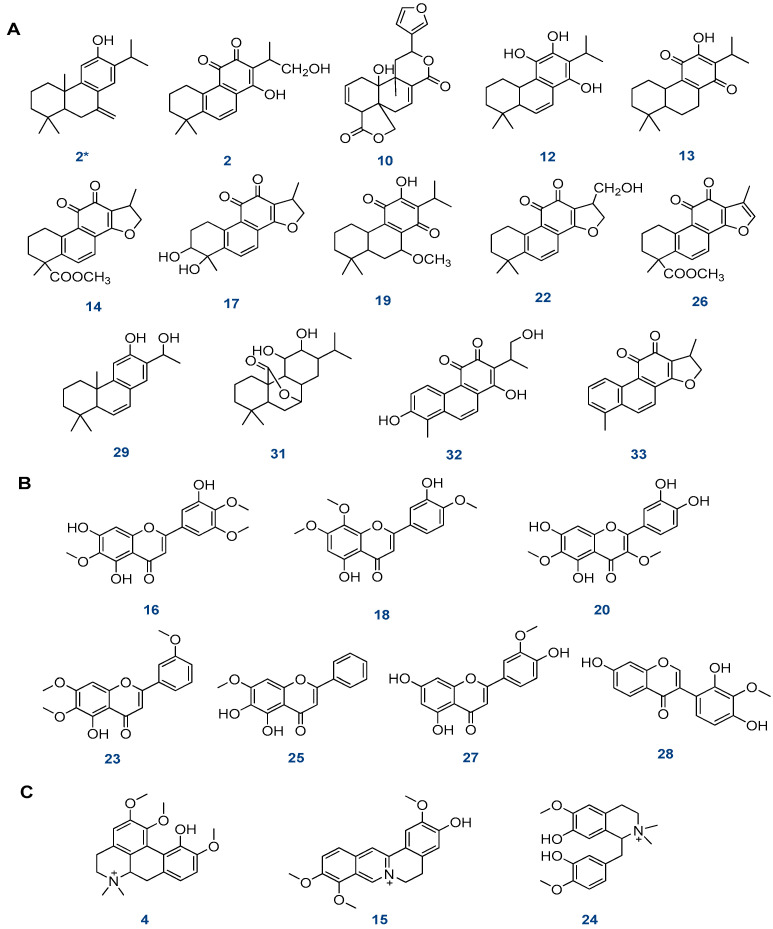
The structure of the tentatively identified compounds in the dichloromethane fraction of *S. hispanica* L. aerial parts. (**A**) Diterpenes, (**B**) flavonoids, (**C**) alkaloids.

The dichloromethane fraction is high in diterpenoids (Figure 3A), most of which are abietane quinones. There were 13 diterpenoids compounds tentatively identified as follows.

**Figure 4:**
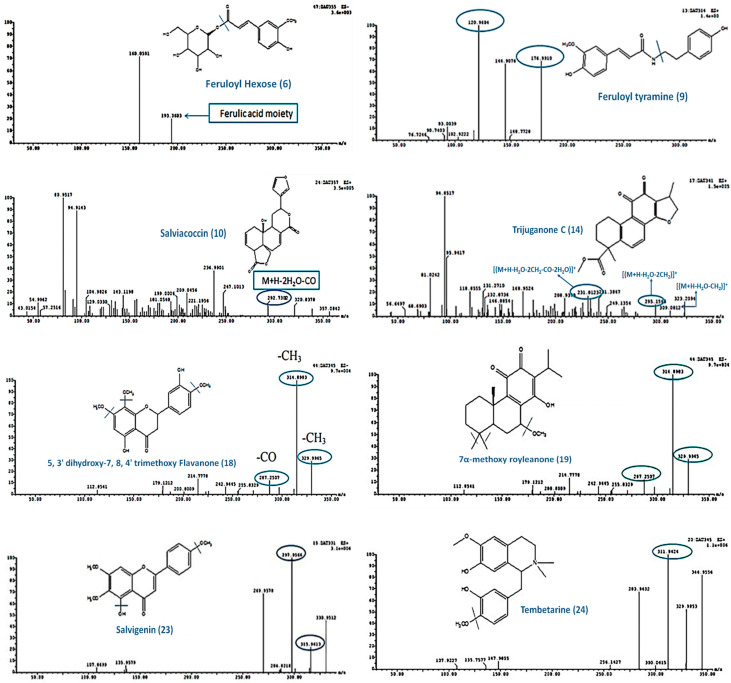
ESI-MS/MS spectrum of some compounds from the dichloromethane fraction of *S. hispanica* L. aerial parts.

Compound **2** (R_t_, 7.26 min) exhibited a precursor ion at *m*/*z* 317 [M+H]^+^ as well as fragment ions at *m*/*z* 299 [(M+H-H_2_O)]^+^ and 267 [(M+H-2H_2_O-CH_2_)]^+^,which are characteristic of tanshinone V [26]. Compound **10** (R_t_, 10.74 min) exhibited a precursor ion at *m*/*z* 357 [M+H]^+^ as well as fragment ions at *m*/*z* 293 [(M+H-2H_2_O-CO)]^+^ and 181. Accordingly, the compound was identified as salviacoccin [20] (Figure 4).

In negative ion mode, compounds **12** and **13** (R_t_, 11.33 and 11.34 min) showed a molecular ion peak at *m*/*z* 315 [M-H]^−^. In the case of **12**, the fragmentation pattern exhibited a fragment ion at *m*/*z* 285 corresponding to [(M+H˗H_2_O-CH_2_)]^+^, but in the case of compound **13**, a fragment ion at *m*/*z* 243 was formed after the loss of [(M+H˗3CH_3_-C_2_H_5_)]^+^. The fragmentation patterns are characteristic of cryptanol and royleanone, respectively [20]. 

Compound **14** (R_t_, 11.44 min) exhibited a precursor ion at *m*/*z* 341 [M+H]^+^ as well as fragment ions at *m*/*z* 309 [(M+H-H_2_O-CH_2_)]^+^, 295 [(M+H-H_2_O-2CH_2_)]^+^, and 231 [(M+H-H_2_O-2CH_2_-CO-2H_2_O)]^+^,which were formed after the loss of C_3_H_6_O. Accordingly, the compound was tentatively identified as trijuganone C [22] (Figure 4). 

Compound **17** (R_t_, 12.27 min) exhibited a precursor ion at *m*/*z* 313 [M+H]^+^ as well as fragment ions at *m*/*z* 249 [(M+H-2H_2_O-CO)]^+^ and 193 [(M+H-2H_2_O-3CO)]^+^. The compound was tentatively identified as tanshindiol C [35]. Compound **19** (R_t_, 12.51 min) showed a precursor ion at *m*/*z* 345 [M-H]^−^, as well as fragment ions at *m*/*z* 330 [(M-H˗CH_3_)]^−^, 315 [(M-H˗2CH_3_)]^−^, and 287 [(M-H˗2CH_3_˗CO)]^−^. The compound was tentatively identified as 7α-methoxy royleanone [37] (Figure 4).

Compound **22** (R_t_, 13.48 min) exhibited a precursor ion at *m*/*z* 313 [M+H]^+^ as well as fragment ions at *m*/*z* 249 [(M+H-2H_2_O-CO)]^+^ and 197. This compound was tentatively identified as hydroxy cryptotanshinone [39].

Compound **26** (R_t_, 14.95 min) exhibited a precursor ion at *m*/*z* 339 [M+H]^+^ as well as a fragment ion at *m*/*z* 311 [(M+H-CO)]^+^, which is characteristic of methyl tanshinonate [35]. Compound **29** (R_t_, 15.51 min) exhibited a precursor ion at *m*/*z* 299 [M-H]^−^ as well as a fragment ion at *m*/*z* 227 [M-H-3CH_3_-C_2_H_3_]^−^. Thus, the compound (**29**) was identified as 16-hydroxy-6,7-didehydroferruginol [20].

Compound **31** (R_t_, 17.04 min) produced both a precursor ion at *m*/*z* 329 [M-H]^−^ as well as a fragment ion at *m*/*z* 285 [(M-H˗CO_2_)]^−^. This fragmentation is typical for carnosol [45].

Compound **32** (R_t_, 25.32 min) showed a precursor ion at *m*/*z* 313 [M+H]^+^, and the presence of a fragment ion at *m*/*z* 269 [(M+H-CO_2_)]^+^ is characteristic of hydroxy tanshinone VI [33]. Compound **33** (R_t_, 27.12 min) exhibited a precursor ion at *m*/*z* 279 [M+H]^+^ and a fragment ion at *m*/*z* 261 [(M+H˗H_2_O)]^+^, and it was identified as 15,16-dihydrotanshinone I [35].

Moreover, seven flavonoid aglycones were tentatively identified in the dichloromethane fraction (Figure 3B), including compound **16** (Rt, 11.99 min), which showed a molecular ion peak at [M-H]^−^ at *m*/*z* 359, as well as fragment ions at *m*/*z* 344, 329, and 314, due to successive losses of CH_3_, and a fragment ion at *m*/*z* 195 that formed after cleavage of the flavone skeleton. Based on this result, the compound was classified as 5,7,3′-trihydroxy-6,4′,5′-trimethoxy flavone [34].

Compound **18** (Rt, 12.47 min) showed a molecular ion peak at *m*/*z* 345 [M-H]^−^; the fragment ions formed after the loss of CH_3_ groups were at *m*/*z* 330, 315, and 287, indicating that **18** could tentatively be identified as 5,3′-dihydroxy-7,8,4′-trimethoxy flavanone [36] (Figure 4). 

Compound **20** (Rt, 12.52 min) showed a molecular ion peak at *m*/*z* 345 [M-H]^−^ in addition to fragment ions formed after successive losses of CH_3_ groups at *m*/*z* 330 and 315. The compound was classified as axillarin (methylated flavonol) [38].

Compound **23** (Rt, 13.69 min) presented an [M+H]^+^ ion at *m*/*z* 331.The MS^2^ spectrum showed fragment ions at *m*/*z* 316 [331-CH_3_]^+^ and *m*/*z* 298 that formed after the loss of H_2_O. The compound was classified as salvigenin (flavone) [40] (Figure 4).

Compound **25** (Rt, 14.93 min) exhibited a sorbifolin (flavone)-specific molecular ion peak at *m*/*z* 301 [M+H]^+^ and a fragment ion at *m*/*z* 286 [41].Compounds **27** and **28** (Rt, 15.21 and 15.40 min) showed identical molecular ion peaks at *m*/*z* 299 [M-H]^−^ in negative ion mode. In the case of compound **27**, the fragment ions at *m*/*z* 284 and 283 were characteristic of diosmetin or chryseriol (flavone) [42], whilst compound **28** revealed fragment ions at *m*/*z* 284 and 255, characteristic of 3′-*O*-methylorobol or gliricidin (isoflavone) [43].

Three alkaloids were tentatively identified from the dichloromethane fraction of aerial parts (Figure 3C), including compound **4** (Rt, 8.82 min), which exhibited a precursor ion at *m*/*z* 357 [M+H]^+^ as well as a fragment ion at *m*/*z* 311 [(M^+^˗CH_3_)_2_ NH)]^+^; this fragmentation is characteristic of menisperine (M^+^:356.4) [28]. Compound **15** (Rt, 11.78 min) exhibited a precursor ion at *m*/*z* 339 [M+H]^+^ as well as a fragment ion at *m*/*z* 295 that was formed after the loss of CH_3_ and CO. Accordingly, jatrorrhizine (M^+^:338.4) was tentatively identified as this compound [28]. Compound **24** (Rt, 14.50 min) showed a fragment ion at *m*/*z* 345 [M+H]^+^. The MS^2^ spectrum showed the fragment ion at *m*/*z* 312 (M+H˗CH_3_OH)]^+^, so the compound was tentatively identified as tembetarine (M^+^:344.4) [28] (Figure 4).

**Figure 5:**
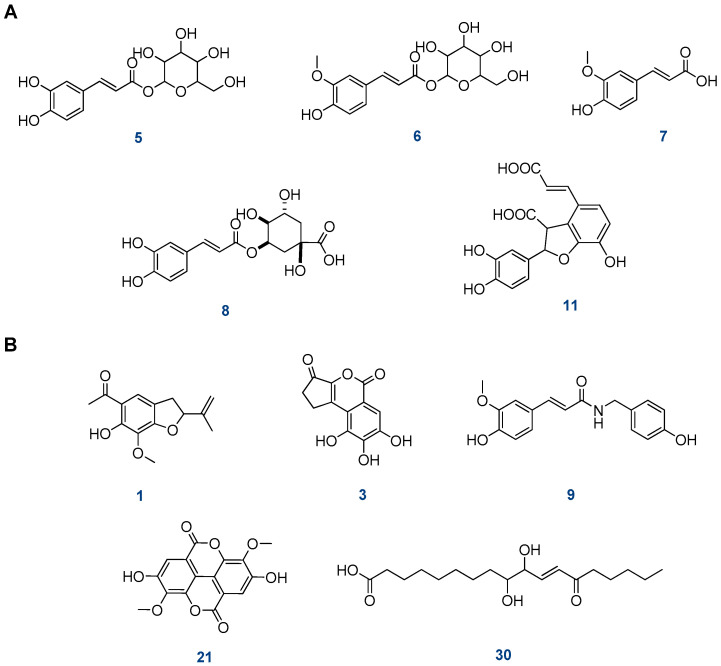
The structure of the tentatively identified compounds in the dichloromethane fraction of *S. hispanica* L. aerial parts. (**A**) Phenolic acid derivatives, (**B**) miscellaneous compounds.

Furthermore, five compounds of phenolic acids and their derivatives were tentatively identified from the dichloromethane fraction of the aerial parts of *S. hispanica* L. (Figure 5A) and are described as follows:

Compound **5** (Rt, 9.00 min) showed a precursor ion at *m*/*z* 343 [M+H]^+^ that was successively subjected to the loss of the hexose sugar moiety to form a fragment ion at *m*/*z* 181 [caffeic acid+H]^+^. Therefore, the compound (**5**) was identified as caffeic acid hexoside [29].

Compound **6** (Rt, 9.12 min) revealed a precursor ion [M-H]^−^ at *m*/*z* 355 and a fragment ion at *m*/*z* 193, corresponding to the ferulic acid moiety after losing hexose sugar. This fragmentation is characteristic of feruloyl hexose [30] (Figure 4). Compound **7** (Rt, 9.96 min) showed a precursor ion at *m*/*z* 195 [M+H]^+^ as well as a fragment ion at *m*/*z* 180 [(M+H-CH_3_)]^+^ and 177 [(M+H-H_2_O)]^+^ after losing CH_3_ and H_2_O, respectively. This fragmentation pattern is characteristic of ferulic acid [31]. Compound **8** (Rt, 10.16 min) exhibited a fragment ion [M-H]^−^ at *m*/*z* 353 in addition to a fragment ion at *m*/*z* 191, corresponding to quinic acid, after losing the caffeoyl moiety. Accordingly, **8** was identified as caffeoyl quinic acid [8]. Compound **11** (Rt, 11.10 min) exhibited mainly a precursor ion at *m*/*z* 359 [M+H]^+^ and a fragment ion at *m*/*z* 315 [M+H-CO_2_]^+^, which is characteristic of przewalskinic acid [33].

Other identified miscellaneous compounds (Figure 5B) were compounds **1** and **3** (Rt, 6.39 and 7.87 min) which exhibited identical precursor ions at *m*/*z* 249 [M+H]^+^. For compound **1**, the fragment ion at *m*/*z* 137 was characteristic of 6-hydroxy, 7-methoxy tremetone, while compound **3** exhibited fragment ions at *m*/*z* 193 [M+H˗2CO]^+^ and *m*/*z* 175 (M+H˗2CO-H_2_O)^+^, characteristic of brevifolin [25,27]. Compound **9** (Rt, 10.53 min) exhibited a precursor ion at *m*/*z* 314 [M+H]^+^ as well as fragment ions at *m*/*z* 177,which corresponded to ferulic aldehyde, and 121,which corresponded to 4-ethylphenol. Thus, compound **9** was tentatively identified as feruloyl tyramine [32] (Figure 4). Compound **21** (Rt, 13.38 min) showed a fragment ion [M-H]^−^ at *m*/*z* 329. The MS^2^ spectrum showed fragment ions at *m*/*z* 314 [(MH˗CH_3_)]^−^, 299 [(M-H˗2CH_3_)]^−^, and 271 [(M-H˗2CH_3_˗CO)]^−^. This compound was identified as dimethyl-*O*-ellagic acid [27]. Compound **30** (Rt, 15.57 min) showed a fragment ion at *m*/*z* 327 [M+H]^+^. The MS^2^ spectrum showed fragment ions at *m*/*z* 229, 211, and 171. The compound was tentatively identified as 13-Oxo-9,10 dihydroxy-11-octadecenoic acid [44].

### 2.2. Isolated Compounds from the Light Petroleum Fraction

**Figure 6:**
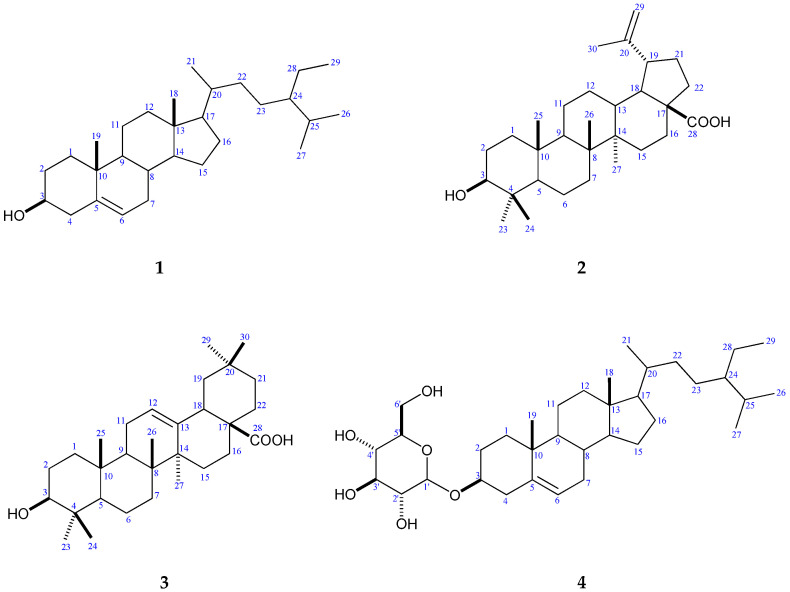
Chemical Structures of isolated compounds (**1**–**4**).

Compounds **1**–**4** were identified as β-sitosterol, betulinic acid, oleanolic acid, and β-sitosterol-3-*O*-β-D-glucoside, respectively, through spectral analyses and comparison with the literature data [18,46,47,48,49], as represented in Figure 6 and Table 3.

β-sitosterol (**1**): white needles; m.p. 137–139 °C; IR (KBr ν_max_, cm^−1^): 3416 (O-H), 2932 and 2864 (C-H aliphatic), 1642 (C=C), 1463 (-CH_2_), 1376 (-CH_3_), and 1051 (C-O). EI-MS: *m*/*z* (relative abundance %) = 414 (M^+^, 100), 399 (24.19), 397 (13), 396 (31.88), 381 (15.54), 367 (1.19), 329 (6.7), 303 (2.88), 119 (1.3), 109 (1.36), 107 (3), 105 (3.41), 95 (5.23), 69 (17.8), 57 (20.9), 55 (17.41), and 43 (27.34). ^1^H- and ^13^C-NMR (CDCl_3_) spectral data are summarized in Table 3.

Betulinic acid (**2**): white amorphous powder; IR (KBr ν_max_, cm^−1^): 3450 (O-H), 2939 and 2867 (C-H), 1682 (C=O), 1642 (C=C), 1449 (CH_2_), 1376 (CH_3_), and 1042 (C-O); EI-MS: *m*/*z* (relative abundance %) = 456 (M^+^, 32), 248 (17.8), 233 (21.3), 220 (100), 207 (14.8), 203 (27.7), 189 (45.7), 175(60.2), 147 (69.8), 91 (18.6), and 79 (19.3). ^1^H-NMR (CDCl_3_) data are summarized in Table 3.

Oleanolic acid (**3**): white amorphous powder; IR (KBr ν_max_, cm^−1^): 3391 (O-H), 2930 (C-H aliphatic), 1687 (C=C), 1458 (CH_2_), 1377 (-CH_3_), and 1023 (C-O); EI-MS: *m*/*z* (relative abundance %) = 456 (M^+^,100), 248 (75.79), 207 (10.48), 203 (17.13), 189 (6.67), and 119 (15.77). ^1^H- and ^13^C-NMR (CDCl_3_) readings are summarized in Table 3.

β-sitosterol-3-*O*-β-D-glucoside (**4**): white crystals; m.p. 272–274 °C; IR (KBr ν_max_, cm^−1^): 3391 (O-H), 2931 and 2866 (C-H aliphatic), 1461 (CH_2_), 1366 (CH_3_), 1069 (C˗O). ESI-MS: *m*/*z* (Relative abundance %) = 577 (M+H^+^, 14.3), 576 (M^+^, 100), 415 (M+H–Glu, 8.89), 267 (20.1), and 211 (34.2). ^1^H- NMR signals of glucose moiety at *δ*(ppm): *δ* 4.21 (1H, d, *J*=10 Hz, H-1‘), *δ* 2.89 (1H, m, H-2‘), *δ* 3.12 (1H, m, H-3‘), *δ* 3.01 (1H, m, H-4‘), *δ* 3.05 (1H, m, H-5‘), δ 3.46 (1H, m, H-6‘b), and *δ* 3.60 ppm (1H, m, H-6‘a).^13^C-NMR signals of glucose moiety at *δ* (ppm): *δ* 101.27 (C-1‘), *δ* 73.94 (C-2‘), *δ* 77.41 (C-3‘), *δ* 70.58 (C-4‘), *δ* 77.21 (C-5‘), and *δ* 61.57 (C-6‘). ^1^H- and ^13^C-NMR (CDCl_3_) data are summarized in Table 3.

### 2.3. GLC-MS Analysis of Seeds Oil

The major fatty acids identified as methyl esters were linoleic acid (35.64%), linolenic acid (23.95), palmitic acid (14.12%), stearic acid (7.63%), lauric acid (5.87%), myristic acid (2.31%), 11,14,17-eicosatrienoic acid (0.59%), arachidic acid (0.57%), caprylic acid (0.54%), and capric acid (0.42%). Polyunsaturated fatty acids (PUFAs) represented 60% of seeds’ oil, while omega-3 fatty acids (linolenic acid) represented 35.64% of the total fatty acids in the seed oil.

### 2.4. Cytotoxic Activity

**Figure 7:**
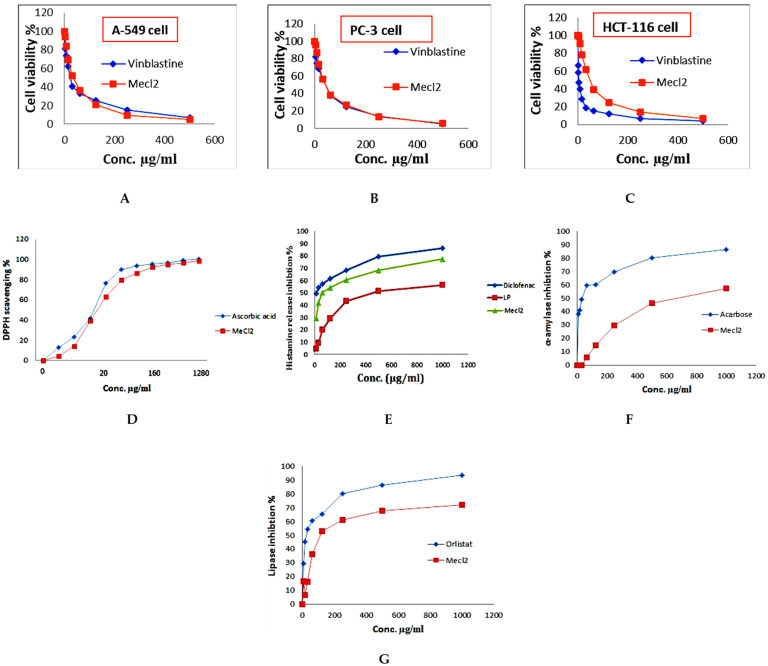
Biological actions of non-polar fractions of *S. hispanica* L. aerial parts. (**A**–**C**) cytotoxic activity, (**D**) antioxidant activity, (**E**) anti-inflammatory activity, (**F**) antidiabetic activity, (**G**) antiobesity activity.

The cytotoxic activity of the dichloromethane fraction was tested using a viability assay with vinblastine as a standard against human lung cancer cell line (A-549), human prostate carcinoma (PC-3), and colon carcinoma (HCT-116). The presence of flavonoids, phenolic compounds, tannin, and glycosides is responsible for cytotoxic activities [50]. The results revealed that the fraction had a moderate cytotoxic activity against A-549, PC-3, and HCT-116 cell lines with IC_50_ of 35.9 ± 2.1 μg/mL, 42.4 ± 2.3 μg/mL, and 47.5 ± 1.3 μg/mL, respectively, and when compared with vinblastine sulfate as a positive control, the IC_50_was 24.6 μg/mL, 42.4 μg/mL, and 3.5 μg/mL, respectively (Figure 7A–C).

### 2.5. Antioxidant Activity

The promising antioxidant result of the dichloromethane fraction refers to the flavonoids and phenolic contents. The hydroxyl groups in phenolic compounds are responsible for antioxidant activity because of their radical-scavenging properties [51]. The DPPH scavenging percentage of the dichloromethane fraction (IC_50_ = 14.73 µg/mL) was approximately comparable to that of ascorbic acid (IC_50_ = 12.50 µg/mL, as shown in Figure 7D.

### 2.6. Anti-Inflammatory Activity

The dichloromethane fraction showed stronger anti-inflammatory activity than the light petroleum fraction, with IC_50s_ of 61.8 μg/mL and 458.6 μg/mL, respectively, compared to diclofenac sodium as a positive control, with IC_50_ of 17.9 μg/mL (Figure 7E). The contents of diterpenes and phenolics in the dichloromethane fraction play important roles in anti-inflammatory activity [52]; sterols, such as β-sitosterol, betulinic acid, oleanolic acid, and β-sitosterol-3-*O*-β-D-glucoside, are also known to exhibit anti-inflammatory activity [53].

### 2.7. Antidiabetic Activity

The antidiabetic activity of the dichloromethane fraction was tested using the α amylase enzyme and acarbose as a positive standard. The results showed that the dichloromethane fraction inhibited the α-amylase enzyme, with IC_50_ of 673.25 μg/mL compared to acarbose, which showed IC_50_ of 34.71 μg/mL (Figure 7F)*. S. hispanica* contains a high concentration of omega-3 fatty acids (35.64% of total fatty acid content), which have been shown to reduce insulin resistance [54].

### 2.8. Antiobesity Activity

There are numerous reports on the antiobesity activity of *S. hispanica* L. seeds but none on the activity of the aerial parts. The antiobesity activity was determined using a pancreatic lipase inhibitory assay, and the results showed that the dichloromethane fraction has moderate antiobesity activity, with IC_50_ 59.3 μg/mL, versus orlist, with IC_50_ 23.8 μg/mL (Figure 7G). The antiobesity activity is due to the presence of poly phenolics, flavonoids, and terpenoids [55].

## 3. Material and Methods

### 3.1. Instruments for Spectroscopic Analyses

Infrared spectral analysis was recorded using the potassium bromide disk technique on a PyeUnicam SP 3000 and IR spectrophotometer of Alpha (I-00523), Jasko, FT/IR-460 plus, Japan. Mass spectra were obtained on Shimadzu GC-MS-QP5050A mass spectrometer at 70 eV. ^1^H and ^13^C-NMR spectral analyses were carried out at the faculty of pharmacy, Ain Shams University, Egypt, using Bruker (Zurich, Switzerland) at 400 and at 100 MHz, respectively. Chemical shifts were given in ppm with the TMS as the internal standard.

### 3.2. Plant Material

*Salvia hispanica* L. aerial parts were collected at the flowering stage from Mushtohor farm (Tokh, Egypt) in March 2018. This plant was identified and verified by Dr. Hussein Abdelbaset (Professor of Plant Taxonomy, Faculty of Science, Zagazig University). A voucher specimen (Lam.S-10) was deposited in the herbarium of the pharmacognosy department, faculty of pharmacy, Zagazig University, Egypt.

### 3.3. Extract Preparation

The air-dried powdered aerial parts of *Salvia hispanica* L. (3 kg) were extracted by cold maceration (5 times × 7 L) using 70% aqueous ethanol. The total extract was evaporated under reduced pressure at 50 °C, yielding 540 gm of dark green viscous residue. The residue (400 gm) was dissolved in a methanol: water mixture (1:9) then subjected to fractionation using light petroleum and dichloromethane. The fractions were washed with distilled water and dried over anhydrous sodium sulfate, then the solvent of each fraction was distilled off under reduced pressure at 50 °C to yield a light petroleum fraction (68 gm) and a dichloromethane fraction (4 gm).

### 3.4. Chromatographic Investigations

The light petroleum fraction was investigated by normal phase TLC using dichloromethane and methanol 99:1. The TLC plates were visualized with anisaldehyde and sulfuric acid, and the promising fractions were subjected to chromatographic investigations.

The light petroleum fraction (33 gm) was chromatographed on a silica gel column packed with light petroleum, and the polarity was increased successively by dichloromethane followed by methanol. Similar fractions were collected according to the TLC profile. Fractions (26–35) eluted by 80% CH_2_Cl_2_/light petroleum were combined, concentrated, and crystallized to obtain four compounds (**1**–**4**).

### 3.5. LC/MS Instrument and Separation Technique

Each fraction (100 μg/mL) solution was prepared using HPLC analytical-grade solvent MeOH, filtered with a membrane disc filter, and then subjected to LC-ESI-MS analysis. Fractional injection volumes (10 μL) were injected into the UPLC instrument equipped with a reverse-phase C-18 column (ACQUITY UPLC—BEH C_18_ 1.7 µm particle size—2.1 × 50 mm column). The mobile phase was prepared by filtering solvents using a filter membrane disc and degassing by sonication before injection. The flow rate was 0.2 mL/min with a gradient mobile phase comprising two eluents: H_2_O acidified with 0.1% formic acid and MeOH acidified with 0.1% formic acid. The parameters for analysis were carried out using positive ion mode as follows: source temperature 150 °C, cone voltage 30 eV, capillary voltage 3 kV, desolvation temperature 440 °C, cone gas flow 50 L/h, and desolvation gas flow 900 L/h. Mass spectra were detected in the ESI between *m*/*z* 100 and 1000. The peaks and spectra were processed using Maslynx 4.1 software and tentatively identified by comparing their retention time and mass spectrum with the reported data.

### 3.6. GLC-MS of Salvia Seeds’ Oil

The seeds were pressed using the Ixtaina et al. method [56], and the oil was derivatized using the Metcalfe et al. method [57] and recorded using Shimadzu GCMS-QP2010 (Tokyo, Japan) equipped with Rtx-1MS fused bonded column and a split–splitless injector. The initial column temperature was kept at 45 °C for 2 min (isothermal), programmed to 300 °C at a rate of 5 °C/min, and kept constant at 300 °C for 5 min (isothermal). The injector temperature was 250 °C. The helium carrier gas flow rate was 1.41 mL/min. All the mass spectra were recorded under the following conditions: (equipment current) filament emission current, 60 mA; ionization voltage, 70 eV; ion source, 200 °C. A series of hydrocarbon samples (1% *v*/*v*) were injected in split mode (split ratio 1:15). The components were identified by matching the retention indices and mass spectra with those reported in NIST17-1 libraries and literature.

### 3.7. Cytotoxic Activity

The anti-cancer activity was carried out using a cell viability assay [58]. Briefly, the cell lines used were the human lung cancer cell line (A-549), human prostate carcinoma cells (PC-3), and colon carcinoma cells (HCT-116), and they were obtained from VACSERA company (Tissue Culture Unit), Cairo, Egypt) [59,60]. The dichloromethane fraction was used in various concentrations (500 to 0 μg/mL). The IC_50_ values of the fractions and the standard (vinblastine sulfate) were calculated.

### 3.8. Antioxidant Activity

The antioxidant activity was determined using the DPPH method according to the Leaves et al. method [61]. Briefly, the dichloromethane fraction was used at different concentrations, 2.5, 5, 10, 20, 40, 80, 160, 320, 640, and 1280 μg/mL, which were each added to 3 mL of DPPH solution, and the decrease in absorbance at 515 nm was determined continuously, with data being recorded at 1min intervals until the absorbance stabilized (16 min). The 50% inhibitory concentrations (IC_50_) of the dichloromethane fraction and the standard (ascorbic acid) were determined.

### 3.9. Anti-Inflammatory Activity

In vitro histamine release assay was performed on light petroleum and dichloromethane fractions according to Venkata et al.’s assay [62]. The results were expressed as inhibition percentage, which was calculated using the following formula:

Inhibitory activity (%) = (1 − As/Ac) × 10

As is the absorbance in the presence of the test substance and Ac is the absorbance of the control substance. The IC_50_ value in μg/mL was estimated.

### 3.10. Antidiabetic Activity

The α-amylase inhibition method was used to determine the antidiabetic activity [63]. Briefly, 1 mL of the dichloromethane fraction of various concentrations (1000 to 7.81 μg/mL) and 1 mL of the enzyme solution were mixed and incubated at 25 °C for 10 min. After incubation, 1 mL of starch (0.5%) solution was added to the mixture and incubated at 25 °C for 10 min. The reaction was then stopped by adding 2 mL of dinitro-salicylic acid, followed by heating the mixture in a boiling water bath for 5 min. After cooling, the absorbance was measured colorimetrically at 565 nm, and the IC_50_ values of the dichloromethane fraction and the standard (acarbose) were estimated.

### 3.11. Antiobesity Activity

The antiobesity activity was determined by pancreatic lipase inhibitory assay [64]. Briefly, the dichloromethane fraction at different concentrations (1000 to 7.81 μg/mL) was pre-incubated with 100 µg/mL of lipase for 10 min at 37 °C. The reaction was then started by adding 0.1 mL of *p*-nitrophenyl butyrate substrate after incubation at 37 °C for 15 min. The amount of *p*-nitrophenol released in the reaction was measured using a multiplate reader (Sigma Aldrich, Burlington, Massachusetts, USA). The IC_50_ values of the dichloromethane fraction and the standard (orlistat) were determined.

## 4. Conclusions

This study represents the first report on the phytochemical constituents of the non-polar fraction of *S. hispanica* aerial parts cultivated in Egypt as well as their pharmacological potentials. The UPLC-ESI-MS/MS analyses of the non-polar fractions (light petroleum and dichloromethane fractions) resulted in the tentative identification of 42 compounds of different chemical classes, including fatty acids, steroids, di- and tri-terpenoids, flavonoids, phenolic acids, and alkaloids. The phytochemical investigation of the light petroleum fraction resulted in the isolation of four compounds, including β-sitosterol (**1**), betulinic acid (**2**), oleanolic acid (**3**), and β-sitosterol-3-*O*-β-D-glucoside (**4**). The GLC-MS analysis of the seeds’ oil revealed that seeds contain a high concentration of omega-3 fatty acids, with a percentage of 35.64% of the total fatty acids content.

Biologically, the dichloromethane fraction showed moderate cytotoxic activity against the human lung cancer cell line (A-549), human prostate carcinoma (PC-3), and colon carcinoma (HCT-116). It also exhibited remarkable antioxidant results that can be attributed to its contents of polyphenolic compounds, in addition to antidiabetic, antiobesity, and anti-inflammatory activities, which are attributed to the fatty acids, steroids, terpenoids, flavonoids, and phenolic acid contents.

In conclusion, these data are considered an addition to the bibliographic data about chia and a contribution towards the exploration of its chemical diversity as well as nutritional and therapeutic value. Henceforth, further studies should be focused towards the isolation of the active principles of the dichloromethane fraction and studying their efficacy, the exact mechanism(s), and safety, which could aid in the development of a new therapeutic agent and/or using chia as a safe natural alternative therapy and nutritional strategy for the treatment of diabetes and obesity in addition to its use as an excellent source of omega-3 fatty acids.

## Figures and Tables

**Table 1:** Tentatively identified compounds in the light petroleum fraction of *S. hispanica* L. aerial parts.

No.	Type	R_t_	M^+^	[M+H]^+^	MS^2^ Fragments	Compound Name	Ref.
1	Steroid	23.57	576	577	415,267,211	β-sitosterol-*O*-glucoside	[18]
2	Diterpene	23.76	300	301	227	Sugiol	[19]
3	Triterpenoid	23.77	278	279	301,279,261	7α-hydroxy-14,15-dinorlabd-8(17)-en-13-one	[20]
4	Steroid	24.13	414	415	414,396,381	β-sitosterol	[21]
5	Fatty acid	24.68	280	281	-	Linoleic acid	[22]
6	Fatty acid	26.54	278	279	-	Linolenic acid	[22]
7	Triterpenoid	29.68	456	457	248,207,203,189,175	Betulinic acid	[23]
8	Triterpenoid	29.68	456	457	248,207,203,189	Oleanolic acid	[24]
9	Fatty acid	29.69	256	257	-	Palmitic acid	[22]

**Table 2:** Tentatively identified compounds in the dichloromethane fraction of *S. hispanica* L. aerial parts.

No.	Type	R_t_	M^+^	[M-H]^−^	[M+H]^+^	MS^2^ Fragments	Compound Name	Ref.
1	Tremetone	6.39	248		249	137	6-hydroxy-7-methoxy Tremetone	[25]
2	Diterpene	7.26	316		317	299,267	Tanshinone V	[26]
3	Coumarin	7.87	248		249	193,175	Brevifolin	[27]
4	Alkaloid	8.82	356		357	311	Menisperine	[28]
5	Phenolic acid	9.00	342		343	181	Caffeic acid hexoside	[29]
6	Phenolic acid	9.12	356	355		193,160	Feruloyl hexose	[30]
7	Phenolic acid	9.96	194		195	180,177,136	Ferulic acid	[31]
8	Phenolic acid	10.16	354	353		191	Caffeoylquinic acid	[8]
9	Phenolic acid	10.53	313		314	177,149,145,121	N-*trans*-Feruloyltyramine	[32]
10	Diterpene	10.74	356		357	293,181	Salviacoccin	[20]
11	Phenolic acid	11.10	358		359	315	Przewalskinic acid	[33]
12	Diterpene	11.33	316	315		299,285	Cryptanol	[20]
13	Diterpene	11.34	316	315		243	Royleanone	[20]
14	Diterpene	11.44	340		341	309,295,231	Trijuganone C	[22]
15	Alkaloid	11.78	338		339	295	Jatrorrhizine	[28]
16	Flavonoid	11.99	360	359		344,329,314,195	5,7,3’-Trihydroxy-6,4’,5’-trimethoxy flavone	[34]
17	Diterpene	12.27	312		313	249,193	Tanshinndiol C	[35]
18	Flavonoid	12.47	346	345		330,315,287	5,3’-Dihydroxy-7,8,4’-trimethoxy flavanone	[36]
19	Diterpene	12.51	346	345		330,315	7-α-Methoxy Royleanone	[37]
20	Flavonoid	12.52	346	345		314,299	Axillarin	[38]
21	Phenolic acid	13.38	330	329		249,197	Dimethyl-O-ellagic acid	[27]
22	Diterpene	13.48	312		313	316,298	Hydroxy cryptotanshinone	[39]
23	Flavonoid	13.69	330	329		345,329,312	Salvigenin	[40]
24	Alkaloid	14.50	344		345	286	Tembetarine	[28]
25	Flavonoid	14.93	300		301	311	Sorbifolin	[41]
26	Diterpene	14.95	338		339	284,283	Methyl tanshinonate	[35]
27	Flavonoid	15.21	300	299		284,255	Diosmetin or Chryseriol	[42]
28	Flavonoid	15.40	300	299		227	3’-O-methylorobol or Gliricidin	[43]
29	Diterpene	15.51	300	299		229,211,171	16-Hydroxy-6,7-didehydroferruginol	[20]
30	Fatty acid	15.57	328	327		285	Oxo-dihydroxy-octadecenoic acid	[44]
31	Diterpene	17.04	330	329		269	Carnosol	[45]
32	Diterpene	25.32	312		313	261	Hydroxy tanshinone VI	[33]
33	Diterpene	27.12	278		279		15,16-Dihydrotanshinone I	[35]

**Table 3:** ^1^H (400 MHz) and ^13^C (100 MHz) NMR spectral data of compounds (**1**–**4**) in CDCl_3_.

No.	1		2	3		4	
	^1^H-NMR	^13^C-NMR	^1^H-NMR	^1^H-NMR	^13^C-NMR	^1^H-NMR	^13^C -NMR
1	1.47 (m)	37.3	0.90 (m) 1.68 (m)	1.64 (m)	38.6	0.98 (m) 1.78 (m)	37.3
2	1.56 (m)	31.7	1.63 (m)	1.61 (m)	27.4	1.47 (m) 1.80 (m)	29.2
3	3.50 (m)	71.8	3.20 (m)	3.32 (dd, 6.4, 4.4 Hz)	78.0	3.60 (m)	78.0
4	2.30 (m)	42.3	-	-	39.0	2.10 (t, 13.2 Hz) 2.30 (d, 12.0 Hz)	38.8
5	-	140.7	0.69 (m)	0.76 (m)	55.4	-	140.9
6	5.36 (m)	121.7	1.40 (m) 1.52 (m)	1.53 (m)	18.8	5.33 (s)	121.7
7	2.02 (m)	31.9	1.43 (m)	1.49 (m)	34.0	1.37 (m) 1.95 (d, 8.0 Hz)	29.7
8	1.69 (m)	31.9	-	-	39.1	1.35 (m)	31.9
9	1.56 (m)	50.2	1.23 (m)	1.55 (m)	47.6	0.92 (m)	50.1
10	-	36.5	-	-	38.4	-	38.5
11	1.51 (m)	21.1	1.20 (m) 1.42 (m)	1.03 (m)	23.0	1.42 (m) 1.47 (m)	21.1
12	1.51 (m)	39.8	1.06 (m) 1.61 (m)	5.25 (t, 4.4 Hz)	125.4	1.13 (m) 1.90 (m)	39.4
13	-	42.3	2.20 (m)	-	138.3	-	42.3
14	1.49 (m)	56.8	-	-	41.9	1.19 (m)	56.7
15	1.58 (m)	24.3	1.40 (m) 2.27 (m)	1.61 (m)	27.4	1.02 (m) 1.50 (m)	24.3
16	1.85 (m)	28.3	1.40 (m) 1.98 (m)	1.05 (m)	23.0	1.66 (m) 1.64 (m)	28.3
17	1.45 (m)	56.1	-	-	47.7	1.07 (m)	55.9
18	0.70 (s)	12.0	1.66 (m)	3.18 (m)	39.4	0.65 (s)	11.3
19	1.02 (s)	19.4	3.02 (m)	3.19 (m)	47.0	0.96 (s)	19.4
20	1.58 (m)	36.2	-	-	30.4	1.32 (m)	36.0
21	0.94 (d, 8.0 Hz)	18.8	1.25 (m)	1.61 (m)	36.8	0.84 (d, 8.0 Hz)	19.1
22	0.93 (m)	34.0	1.55 (m) 2.00 (m)	1.30 (m)	33.0	0.81 (m) 1.25 (m)	33.8
23	1.16 (m)	26.1	0.99 (s)	0.99 (s)	27.9	1.12 (m)	25.9
24	1.38 (m)	45.9	0.77 (s)	0.79 (s)	16.7	0.89 (m)	45.6
25	1.56 (m)	29.2	0.85 (s)	0.91 (s)	15.3	1.61 (m)	30.2
26	0.84 (d, 8.4 Hz)	19.8	0.96 (s)	0.84 (s)	17.3	0.89 (m)	20.2
27	0.86 (d, 8.4 Hz)	19.0	1.00 (s)	1.13 (s)	26.5	0.78 (m)	19.6
28	1.10 (m)	23.1	-	-	181.2	1.23 (m) 1.26 (m)	23.1
29	0.82 (m)	12.0	4.63 (s) 4.76 (s)	0.89 (s)	36.7	1.17 (m)	12.1
30	-	-	1.71 (s)	0.98 (s)	24.0	-	-

## Data Availability

Not applicable.
